# Full sequence analysis and characterization of the South Korean Norovirus GII-4 variant CUK-3

**DOI:** 10.1186/1743-422X-8-167

**Published:** 2011-04-14

**Authors:** Jeong-Woong Park, Sung-Geun Lee, Young-Min Lee, Weon-Hwa Jheong, Sangryeol Ryu, Soon-Young Paik

**Affiliations:** 1 Department of Food and Animal Biotechnology, Department of Agricultural Biotechnology, Center for Agricultural Biomaterials, and Research Institute for Agriculture and Life Sciences, Seoul National University, Seoul 151-921, Republic of Korea; 2 Department of Microbiology, College of Medicine, The Catholic University of Korea, Seoul 137-701, Republic of Korea; 3 Department of Microbiology, College of Medicine and Medical Research Institute, Chungbuk National University, Cheongju, Chungcheongbuk-do 361-763, Republic of Korea; 4 Environmental Infrastructure Research Department, National Institute of Environmental Research, Incheon, 404-708, Republic of Korea

**Keywords:** Norovirus, Sequence, Phylogenetic tree

## Abstract

**Background:**

Many of researchers have focused on the emerging pathogen, Norovirus, since its first identification as the causing agent of nonbacterial acute gastroenteritis in humans. One of the virulence factors of norovirus, the great genetic diversity attributed to point mutations and recombinations, has brought forth the result of significant changes in the circulating norovirus genotype patterns.

**Findings:**

In recognition of the necessity for tracking and monitoring of genetic diversity, a norovirus variant among the most prevalent genotype GII-4, Norovirus Hu/GII-4/CUK-3/2008/KR (CUK-3), was isolated from stool samples and analyzed on the level of whole genome sequence. Whole genome sequence analysis revealed three ORF composites of the whole genome, ORF1 (5100 bp), ORF2 (1623 bp), and ORF3 (807 bp). Each genetic relationship of CUK-3 variant analysis located the ORF1 (5,100 bp) in Cluster I, ORF2 (1623 bp) in Cluster I (2006b), ORF3 (807 bp) in Cluster I, and the whole genome sequence (about 5.1 kb) in Cluster I in the phylogenetic tree. And the phylogenetic analyses showed the same location of CUK-3 strain with the GII-4/2006b cluster in the phylogenetic tree.

**Conclusions:**

In This study, a first concerning the full-length sequence of a NoV variant in South Korea is meaningful in that it can be used not only as a full-length NoV variant sequence standard for future comparison studies, but also as useful material for the public health field by enabling the diagnosis, vaccine development, and prediction of new emerging variants.

## Findings

Noroviruses (NoVs) are the most important viruses that cause nonbacterial acute gastroenteritis in humans. In addition to increased susceptibility, the elderly are at increased risk for more severe disease and death, as are the very young and the immunocompromised [1,2]. They are small, and non-enveloped viruses which and belong to the family *Caliciviridae*, genus *Norovirus*. Noroviruses have a single positive-strand NoV RNA genome of about 7.6 kb in size. Three open reading frames (ORFs) have been identified in the NoV genomes. ORF1 encodes a polyprotein that is cleaved into six non-structural (NS) proteins, which carry amino acid sequence motifs conserved in NTPase, protease and RNA-dependent RNA polymerase (RdRp) [3,4]. ORF2 encodes a major structural protein, Viral Protein (VP1), which consist of two domains-the shell domain (S) and the protruding arm (P) that is again divided into two subdomains, P1 and P2. The S domain is highly conserved while the P domain is variable. P2 of the P domain is hypervariable and carries immune and cellular recognition sites [5-7]. ORF3 encodes minor capsid protein, VP2, which is rich in basic amino acids and is proposed to have a role in viral stability [8,9]

Recently, NoVs were recognized as novel emergent pathogens. The main route of transmission is suspected to be person-to-person, but food and water-borne transmission is also important [1,10,11]. According to nucleotide sequence analysis of the capsid regions, noroviruses are classified into five genogroups, GI to GV, each of which can be further divided into several clusters or genotypes [12]. Among the five genogroups, three genogroups (GI, GII and GIV) are known to cause clinical illness in humans, and genotype GII-4 has been the predominant circulating strain to the present [13]. The error-prone RNA replication and recombination between viruses is what drives noroviruses to its the great diversity. Furthermore, the accumulated mutations of the hypervariable P2 domain of the VP1 protein produced different GII-4 NoVs [14]. The most representative of the resulting variant GII-4 strain, GII-4/2006b, with 3 nucleotide insertions in the P2 domain at position 6265, emerged in the summer of 2002 which lead to a major gastroenteritis outbreak as well as an epidemic gastroenteritis worldwide in the winter of 2002/2003 [15,16]. In South Korea, gastroenteritis outbreaks by GII-4/2006b variants have been reported from September of 2007 to July of 2008 had been reported [17].

In this paper, the whole genome sequence of another isolated variant of the emerging strain type, the GII-4 variant, was analyzed and compared with other variants to reveal the genetic relationship and to predict the tendency of GII-4 variants in South Korea.

NoV positive-stool sample was isolated from patients with acute gastroenteritis in Daejeon, South Korea in November 2008. The sample was obtained from the Waterborne Virus Bank (Seoul, Korea). The stool sample was stored at -70°. The viral genomic RNA was extracted from 140 μl of 10% fecal suspension with the QIAamp^® ^Viral RNA Mini kit (Qiagen, Hilden, Germany) in accordance with the manufacturer's instructions.

For the detection of NoV, reverse transcription PCR (RT-PCR) was performed with the One Step RT-PCR kit (Qiagen, Hilden, Germany) with primers based on the sequence of the norovirus capsid region (Table 1).

**Table 1:** Primer sets used in this study

	Primer	Sequence (5'→ 3')	Polarity	**Region**^**a**^
Diagnosis primer sets	GIF1M	CTG CCC GAA TTY GTA AAT GAT GAT	+	5342-5365
	GIRIM	CCA ACC CAR CCA TTR TAC ATY TG	-	5649-5671
	GIIF1M	GGG AGG GCG ATC GCA ATC T	+	5049-5067
	GIIRIM	CCR CCI GCA TRI CCR TTR TAC AT	-	5367-5389

Designed primer sets	ORF1-1F	GTG AAT GAA GAT GGC GTC TAA C	+	1-22
	ORF1-1R	AGT TCC ACT GCA AGG TCC TCA G	-	999-1020
	ORF1-2F	CTG AGG ACC TTG CAG TGG AAC T	+	999-1020
	ORF1-2R	ATG AGG GAA CCA GTG GTG AGA GT	-	2018-2040
	ORF1-3F	CGT GCT CGA GGC GCA TCG ATT T	+	1821-1842
	ORF1-3R	TTG TAC TCA TCA TAC TCT TCA	-	2701-2721
	ORF1-4F	CAT TGC TCG AGC ATC AGG GCT AC	+	2038-2060
	ORF1-4R	TTG ACT ATC CTC GAC CAG ATG CT	-	3038-3060
	ORF1-5F	AGC ATC TGG TCG AGG ATA GTC AA	+	3038-3060
	ORF1-5R	GTG GCA CAT ATG ACA GTG TTT CC	-	3518-3540
	ORF1-6F	GGA AAC ACT GTC ATA TGT GCC AC	+	3518-3540
	ORF1-6R	CAG TCG TTC TTC CGC ATG TGG TGC GG	-	3935-3960
	ORF1-7F	CTC AGC ACC AAG ACT AAA TTC TGG A	+	3635-3659
	ORF1-7R	TGG GCG ATG GAA TTC CAT TGA GAG G	-	4485-4509
	ORF1-8F	ATG GTT AAA TTC TCC CCA GAA CC	+	4355-4377
	ORF1-8R	CCA CCT GCA TAA CCA TTG TAC AT	-	5367-5389
	ORF2-F	ATG AAG ATG GCG TCG AAT GGC	+	5085-5105
	ORF2-R	TTA TAA TGC ACG CCT GCG CCC CGT	-	6681-6704
	ORF3-F	ATG GCT GGA GCT TTC TTT GCT	+	6704-6724
	ORF3-R	AAA GAC ACT AAA GAA AGG AAA GAT	-	7532-7555

^a ^Each of sequence number of primer sets region is indicated with Lordsdale strain (X86557) except that of GIF1M and GIR1M set indicated with Norwalk strains (M87661).

To analyze the whole genome sequence of the detected norovirus strain, reverse transcription PCR (RT-PCR) was performed with the One Step RT-PCR kit (Qiagen, Hilden, Germany) with 10 pairs of newly designed primer sets (Table 1). Ten fragments were amplified; eight fragments for ORF1, one fragment for ORF2, and one fragment for ORF3. The PCR products were then analyzed by electrophoresis on 1.5% agarose gel and ethidium bromide staining. The amplified fragments were purified with HiYield™ Gel/PCR DNA Extraction kit (RBC, Taipei, Taiwan) from the gel. Then, the products were cloned into the pGEM-T Easy vector (Promega, USA) and were sequenced by Genotech (Daejeon, South Korea).

Sequence data analysis of the composite sequence of ten plasmids aligned with Clustal W method using the DNAStar software (DNAStar Inc.) revealed that the whole genome was composed of three ORFs, 5100 bp (ORF1), 1623 bp (ORF2), and 807 bp (ORF3).

The dendrograms were constructed with the neighbor-joining method. The nucleotide sequence data reported in this study have been deposited into GenBank (accession number; FJ514242).

A comparison of the nucleotide and amino acid sequences of the entire ORF2 with reference strains showed that CUK-3 strain had the highest sequence similarity to the NoV GII-4 reference strain X76717. The nucleotide similarity with CUK-3 was 60.1 - 88.0% with the reference strains. A protein database search (BLASTX) for the product of ORF2 yielded 59.3 - 92.0% similarity. However, the cut-off value for amino acid similarity between the CUK-3 variant and the GII-4 reference strains showed low identity (92.0%) for the genomic sequences (Table 2).

**Table 2:** Nucleotide and amino acid sequence similarities between CUK-3 strain and the full length ORF2 sequences from different Genogroup GII reference strains.

Genotype	Strain	**Acession No**.	Similarity (%)	
GII-1	H awaii-USA94	U07611	64.9^a^	64.6^b^
GII-2	Msham-GBR95	X81879	62.8	63.9
GII-3	Toronto-CAN93	U02030	64.8	67.4
GII-4	Bristol-GBR-93	X76716	88.0	92.0
GII-5	Hilingd-GBR00	AJ277607	64.6	63.9
GII-6	Seacrof-GBR00	AJ277620	63.0	63.9
GII-7	Leeds-GBR00	AJ277608	64.3	64.8
GII-8	Amstdam-NLD99	AF195848	64.3	64.8
GII-9	VABeach-USA01	AY038599	63.7	63.5
GII-10	Eufurt-DEU01	AF421118	63.5	64.8
GII-11	SW918-JPN01	AB074893	62.3	64.6
GII-12	Wortley-GBR00	AJ277618	63.3	63.9
GII-13	Faytvil-USA02	AY113106	64.5	64.3
GII-14	M7-USA03	AY130761	64.3	64.8
GII-15	J23-USA02	AY130762	60.1	59.3
GII-16	Tiffin-USA03	AY502010	64.1	65.9
GII-17	CSE1-USA03	AY502009	63.7	65.2

^a ^The nucleotide sequences of the ORF2 regions starting at nucleotide 1620 were analyzed.

^b ^The amino acid sequences of the ORF2 regions starting at amino acid 540 were analyzed.

**Figure 1:**
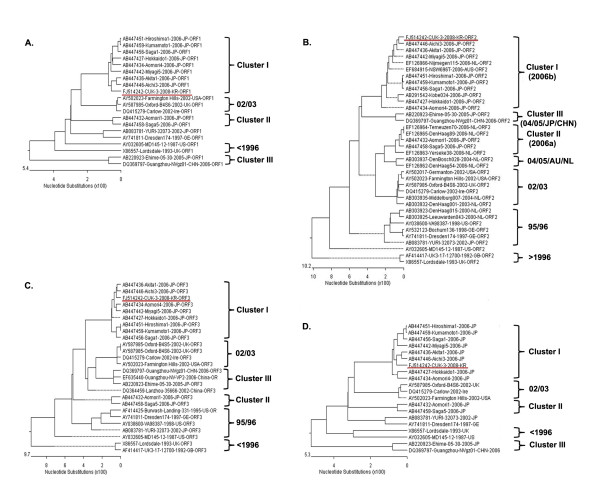
**Phylogenetic trees of the nucleotide sequences of the complete regions of NoV GII/4**. Phylogenetic tree generated based on the complete sequence of the ORF1 (about 5.1 kb) (A), ORF2 (about 1.6 kb) (B), ORF3 (about 0.8 kb) (C), and near-full-length genome (7.5 kb) (D)

To analyze the genetic relationship between the CUK-3 variant and the other variants reported worldwide, the sequences of ORF sequences and the whole genome were processed with multiple sequence alignment. Each analysis of CUK-3 located the ORF1 (5,100 bp) in Cluster I, ORF2 (1623 bp) in Cluster I (2006b), and ORF3 (807 bp) in Cluster I as well as the whole genome sequence (about 5.1 kb) in Cluster I (Figure 1).

As shown on the case analysis of ORF1, Cluster I contains Hiroshoma1, Kumamoto1, Saga1, Hokkaodo1, Aomori4, Miayagi5, Akita1, Aichi3 and showed the highest similarity range (98.0~98.9%) (Figure 1A).

As shown on the case analysis of ORF2, Cluster I (2006b) contains emerged the GII-4 varints in Japan and showed the highest similarity range (97.7 - 99.0%), whereas Cluster II (2006a) showed low similarity range (91.7 - 92.3%) (Figure 1B).

As shown on the case analysis of ORF3, Cluster I showed the highest similarity range (97.9 - 99.3%). Interestingly, 02/03 group contains emerged the GII-4 vaiants in Europe showed more higher similarity range (93.1 - 93.6%) than Cluster II contains emerged the GII-4 variants in Japan (Figure 1C).

As shown on the case analysis of full length, the CUK-3 strain showed the highest similarity (98.3%) with the 2006 epidemic strain, Aichi3/2006/JP strain, whereas Ehime/5-30/2005/JP strain and Lordsdale/1993/UK strain showed the lowest similarity (89.2%) (Figure 1D).

The CUK-3 strain can be placed on the same branch of the phylogenetic tree as the GII-4/2006b cluster, based on the nucleotide sequence of the RdRp region and the amino acid sequence of the capsid protein (data not shown). Also, the analysis of the GDD motif (AANNTG) of the RdRp region of the GII-4 variants reported worldwide indicated that the GDD motif of CUK-3 strain was similar to that of the GII-4 variants of the epidemic strains in 2006. These strains were common in Hong Kong, Japan, and other regions of North East Asia during the same period [12,18]. Such similarities in analyses between CUK-3 and GII-4/2006b strain are evidence of the reemergence of GII-4 variant.

**Figure 2:**
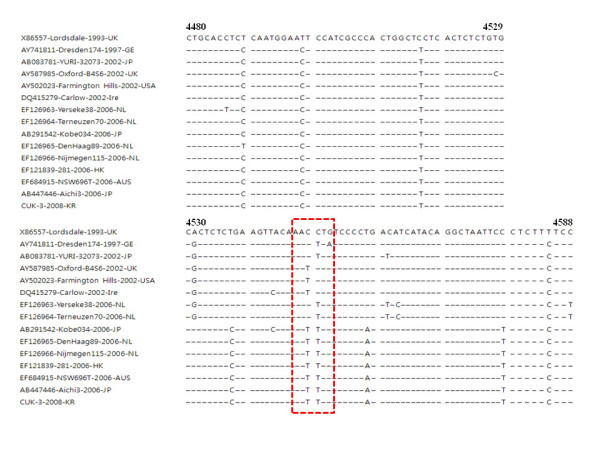
**Partial sequence comparison of the RdRp gene**. The boxed sequence is the conserved GDD motif, ANNNTG. Nucleotide positions are referenced to the Lordsdale-1993-UK sequence.

In Europe and Asia, most norovirus outbreaks that occurred after 2002 have been caused by GII-4 variant strains (Figure 2). In addition, biennial emergences of pandemic NoV GII-4 variants have been reported in 2002, 2004, 2006 and 2008 [12,18,19]. These GII-4 variants have also been identified in South Korea and are rapidly spreading worldwide.

Amino acid sequences of the capsid region encoded by ORF2 was compared with a total of 39 GII-4 variants reported from various countries including US, UK, Japan, China, Germany etc. Date of 540 amino acid analyses showed the pattern of changes of two amino acid in first P1 domain, aa238 (S _GII-4- >2002 _→ T _GII-4- <2002_), aa250 (Y _GII-4- >2002 _→ F _GII-4- <2002_). The P2 domain showed five substitutions of amino acids, aa280 (A _GII-4- >2002 _→ P _GII-4- <2002_), aa346 (A _GII-4- >2002 _→ P _GII-4- <2002_), aa367 (F _GII-4- >1995 _→ Y _GII-4- >2002 _→ F _GII-4- <2002_), aa376 (Q _GII-4- >2002 _→ E _GII-4- <2002_), and aa394 (G or S _GII-4- >2006_→ T_GII-4- <2006_). In addition, the second P1 domain also showed two amino acid substitutions, aa459 (L _GII-4- >1995 _→ Q _GII-4- >2002_) and aa504 (P _GII-4- >2002 _→ Q _GII-4- <2002_). These analytic data showed that there had already been numerous mutations, which lead to the production of a wide range of variants, already before 2002 and produced various variants. And as shown on the case of aa394 of P2 domain which inserted novel amino acid, G or S, and then substituted to T after 2006 (data not shown). Although previous studies have indicated that the sequence for the NoV capsid region is useful for the diagnosis of NoV variants, no studies have analyzed the RdRp region of the South Korean NoV GII-4 variant. This is the first report that describes the full-length sequence of a NoV GII-4 variant that was isolated from clinical samples in South Korea. We suggest that this sequence can be used as a standard for the comparison of full-length NoV GII-4 variant sequence with other strains.

The information acquired from whole genome sequencing in this study can be useful not only for a more accurate diagnoses of NoVs but also for the basic research for the elucidation genetic functions. Furthermore, it will be helpful for the prediction of newly appearing pandemic variants through comparison with GII-4 variants in neighboring countries, fundamental research for vaccine development, and eventually , for the field of public health through with the provision of new emerging strains of NoV.

## Competing interests

The authors declare that they have no competing interests.

## Authors' contributions

SRR and SYP conceived this study. YML, WHJ and designed and conducted the experiments. SGL and JWP analyzed the sequence data and carried out the molecular phylogenetic analysis. SGL and SYP wrote the manuscript. All authors read and approved the final manuscript.
